# The role of irradiance and C-use strategies in tropical macroalgae photosynthetic response to ocean acidification

**DOI:** 10.1038/s41598-018-27333-0

**Published:** 2018-06-21

**Authors:** Regina C. Zweng, Marguerite S. Koch, George Bowes

**Affiliations:** 1 Biological Sciences Department, Aquatic Plant Ecology Lab, Florida Atlantic University, 777 Glades Rd, Boca Raton, FL 33431 USA; 2 Department of Biology University of Florida, 220 Bartram Hall, Gainesville, FL 32611 USA; 3 Present Address: Department of Ecology and Evolutionary Biology, University of California, 618 Charles E Young Dr S, Los Angeles, CA 90095 USA

## Abstract

Fleshy macroalgae may increase photosynthesis with greater CO_2_ availability under ocean acidification (OA) and outcompete calcifying macroalgae important for tropical reef accretion. Macroalgae use energy-dependent carbon concentrating mechanisms (CCMs) to take up HCO_3_^−^, the dominant inorganic carbon for marine photosynthesis, but carbon-use strategies may depend on the *p*CO_2_, pH and irradiance. We examined photosynthesis in eight tropical macroalgae across a range of irradiances (0–1200 μmol photon m^−2^ s^−1^), pH levels (7.5–8.5) and CO_2_ concentrations (3–43 μmol kg^−1^). Species-specific CCM strategies were assessed using inhibitors and δ^13^C isotope signatures. Our results indicate that the log of irradiance is a predictor of the photosynthetic response to elevated *p*CO_2_ (R^2^ > 0.95). All species utilized HCO_3_^−^, exhibited diverse C-use pathways and demonstrated facultative HCO_3_^−^ use. All fleshy species had positive photosynthetic responses to OA, in contrast to a split amongst calcifiers. We suggest that shifts in photosynthetically-driven tropical macroalgal changes due to OA will most likely occur in moderate to high-irradiance environments when CCMs are ineffective at meeting the C-demands of photosynthesis. Further, facultative use of HCO_3_^−^ allows greater access to CO_2_ for photosynthesis under OA conditions, particularly amongst fleshy macroalgae, which could contribute to enhance fleshy species dominance over calcifiers.

## Introduction

The oceans have been absorbing ~30% of the total global anthropogenic CO_2_ emissions emitted annually^1^, representing ~2.9 GtC y^−1^ of the ~10.7 GtC y^−1^. The oceans sequester this excess atmospheric CO_2_ with the resulting consequence of lowering ocean pH, referred to as ocean acidification (OA). Ocean acidification is resulting in a wide-range of impacts on marine organisms and ecosystems sensitive to shifts in the carbonate chemistry^2–7^ (an increase in *p*CO_2_ and HCO_3_^−^ and a decline in CO_3_^2−^). While the effects of elevated atmospheric CO_2_ levels on terrestrial plant photosynthesis has been well studied over the last several decades, our understanding of elevated CO_2_ effects on marine macroalgal photosynthesis has only been emerging over the last decade^8–13^. In marine systems, increased *p*CO_2_ and HCO_3_^−^ levels under OA has the potential to provide dissolved inorganic carbon (DIC) for algal photosynthesis when carbon is limiting. A review of OA studies indicate that raising ocean *p*CO_2_ and lowering pH have negative effects on growth, calcification and metabolism of many calcifying macroalgae, but a positive effect on the growth of non-calcareous, fleshy species^6,7,14^. This is a disturbing trend as many fleshy macroalgae can readily become nuisance species^15^ and outcompete calcifiers important for cementing and accreting reefs. While an increase in fleshy over calcifying macroalgae under OA is recognized as a potential problem^16,17^, the mechanisms accounting for OA-induced growth responses in macroalgal species are elusive. OA-induced growth responses may be related to species-specific photophysiology, as has been found near naturally acidified volcanic seeps or vents^18^, which are considered a useful proxy for long-term exposure to elevated *p*CO_2_ in the field.

Inorganic carbon uptake strategies may be a factor in determining macroalgal responses to elevated *p*CO_2_, because individual species use different mechanisms of inorganic carbon uptake. HCO_3_^−^ use can be advantageous in marine macroalgae because the ocean pH (8.1) maintains CO_2_ in seawater at a low concentration (13 μmol kg^−1^) compared to HCO_3_^−^ (1867 μmol kg^−1^). Furthermore, CO_2_ diffuses 10,000 times slower in water compared to air, thus CO_2_ availability can limit marine macroalgal photosynthesis^5^. Many marine macroalgae depend on HCO_3_^−^ to supplement CO_2_ as a source of inorganic carbon for photosynthesis to overcome inorganic carbon limitation^5,19^. Carbon concentrating mechanisms (CCMs) in macroalgae are characterized by a range of efficiencies in providing CO_2_ to rubisco for photosynthesis^18^. One CCM mechanism is the secretion of external carbonic anhydrase (CA_ext_) into the cell wall where it catalyzes the dehydration of HCO_3_^−^ to CO_2_ after which it can passively diffuse into the cell^20,21^. A second mechanism is the use of ATPase H^+^ pumps that lower the pH at the algal surface and shift the carbonate equilibrium towards CO_2_. This mechanism can also generate a proton-motive force for active transport of CO_2_ or HCO_3_^−^ ^22–24^. So far, evidence for H^+^ pumps has been presented for only a few temperate marine macroalgal species and microalgae^25^ and its linkage to photosynthetic OA response is unknown. Another mechanism is an anion exchange (AE) protein that facilitates the active uptake of HCO_3_^−^ ^26–29^.

Due to the fact that species using HCO_3_^−^ may be less carbon limited under current *p*CO_2_ than species that rely solely on CO_2_, it has been hypothesized that algae which use HCO_3_^−^ and possess CCMs will be less responsive to increases in *p*CO_2_ than species that only use CO_2_ ^6^. This is supported by studies that have shown temperate macroalgae that rely exclusively on CO_2_ have increased growth and photosynthesis under elevated *p*CO_2_ ^22^, whereas macroalgae that utilize HCO_3_^−^ elicit no photosynthetic response to elevated *p*CO_2_ ^30^. Counter to these results, lower isotopic signatures, an indicator of greater CO_2_ use, was more prevalent in macroalgae closer to high CO_2_ seeps in the field, suggesting species that utilize greater CO_2_, when available, may be more competitively dominant, even when they also possess CCMs. These data suggest potential facultative HCO_3_^−^ use may be selected for in a high CO_2_ ocean if CCMs are downregulated^30^, and may confer competitive dominance. While facultative HCO_3_^−^ use may be an optimal carbon use strategy, species in low-light environments can be restricted to non-CCM strategies due to the energy requirements of active C-use mechanisms^31,32^. Thus, establishing the role of C-use mechanisms and light in modulating responses to OA is required to clarify under what conditions photosynthesis has the potential to increase under greater ocean CO_2_ availability.

In this study, we examined if species-specific carbon uptake mechanisms would determine the photosynthetic response of five calcareous and three fleshy tropical macroalgae to elevated *p*CO_2_ and lower pH, including those predicted for 2100^33^ (scenario RCP 8.5). We asked if species employing CCMs are obligate HCO_3_^−^ users or if they respond to greater CO_2_ availability indicative of a facultative, flexible physiology in regard to inorganic carbon sequestration. Stable isotope signatures were used as an indicator of their inorganic carbon source for photosynthesis based on the assumption that macroalgae with δ^13^C values > −10 only use HCO_3_^−^, those between −11 and −30% are both HCO_3_^−^ and CO_2_ users, while those with values < −30% are restricted to CO_2_ use^9,34^. Specific HCO_3_^−^ use mechanisms were assessed using inhibitors. Further, we examined the interactive role of light by assessing the photo-physiological responses to pH across a broad range in irradiances (50 to 1,200 μmol photon m^−2^ s^−1^), comparing photosynthetic efficiency, maximum net/gross photosynthesis, light compensation point and respiration, based on photosynthesis-irradiance curves. We hypothesized that species with the capacity to utilize HCO_3_^−^ for photosynthesis would show little or no enhancement of photosynthesis in response to lowered pH and elevated *p*CO_2._ Based on the dominance of fleshy species at CO_2_ enriched sites in the field^17,35,36^, particularly those in the brown phyla, we proposed that fleshy macroalgae would preferentially increase photosynthesis compared to calcifying species under low pH.

## Methods

### Species and Sampling Sites

Macroalgae were collected from a shallow (~3 m), high irradiance (700–1200 μmol photon m^−2^ s^−1^ bottom) patch reef along the Florida Reef Tract at Looe Key (24°37.233′N, 81°22.247′ W) on five collection trips (May 2016 to January 2017). Field light levels were measured just above the benthos with a 4π spherical PAR quantum sensor (LI-193, LI-COR Inc.). Species included five calcifying and three fleshy species, representing the three macroalgal phyla: calcified green algae (*Halimeda opuntia* and *Udotea luna*), calcified red algae (*Jania adhaerens*, *Neogoniolithon strictum*, assemblage of crustose coralline algae [CCA]), fleshy brown algae (*Sargassum fluitans*, *Canistrocarpus cervicornis* [previously genus *Dictyota*]), and a fleshy red alga (*Laurencia intricata*). With the exception of CCA and *Saragassum*, algae were collected by removing the whole thallus from the substrate. Thalli branches were kept intact when subsampling to minimize disturbance. CCA were collected on 80 small Plexiglas plates (2 cm × 1 cm).

During macroalgal collections, site pH (Orion A211, 8302BNUMD pH meter calibrated with a CRM, Dixon Lab), temperature, irradiance and salinity were determined in the field. Water samples (n = 3; 60 mL) were collected and total alkalinity determined within 48 h (Titrando® Metrohm USA, Inc.; CRM, Dixon Lab at Scripps Institute of Oceanography). Five carboys of seawater were collected to run experiments with seawater from the study site seawater. Alkalinity, temperature, conductivity and pH data were used to calculate DIC speciation (CO_2_ SYS^37^). Macroalgae were transported to the laboratory in an aerated cooler and transferred to aquaria with carbonate sand and seawater from the study site *situ* seawater. Aquaria were kept in a water bath at 27 °C, the average seasonal temperature on the Florida Reef Tract; light was maintained on a 12:12 light/dark cycle (150 μmol photon m^−2^ s^−1^). Salinity and temperature were measured and maintained at ambient levels (~36 psu and 27 °C) throughout the experiment. All experiments were run within two weeks of collection. Replicates for each experiment were run sequentially to account for any differences in responses for algae immediately taken from the field growing at 700–1200 μmol photon m^−2^ s^−1^ and those in the lab maintained at a lower light level; our excellent replication among treatments provides confidence that algal responses were not significantly influenced by short-term exposure to lower irradiance. Further, no photoinhibition was found for any algal species at high experimental irradiance.

### pH Experiment

Photosynthetic and respiration rates were determined at four pH values: high (8.5), ambient (8.1), projected levels for 2100 (7.8 pH, RCP 8.5)^33^ and low (7.5). Different individuals were used for each run (~224 runs total, 8 sp × 6–8 replicates × 4 pH treatments) and runs conducted between 10:00 to 19:00 in filtered (0.45 μm) seawater. To achieve pH treatments, CO_2_ gas was bubbled into seawater to lower pH (7.8 and 7.5) and 0.1 M NaOH was added to raise pH (8.5). The pH meter (Orion A211) was calibrated daily with a pH standard (CRM, Dixon Lab at Scripps Institute of Oceanography). Alkalinity, temperature, conductivity and pH were used to calculate CO_2_ concentrations in each pH treatment (CO_2_ SYS). Alkalinity was 2,369, 2,378, 2,449, and 2,805 μmol kg^−1^ for pH treatments 7.5, 7.8, 8.1, and 8.5 respectively. The higher alkalinity in the high pH treatment was due to adjusting pH with NaOH^38^; however, the change in alkalinity was due to an increase in hydroxyl anions (OH^−^), because no additional carbon was added to the system. The four pH treatments (7.5, 7.8, 8.1 and 8.5) resulted in approximately an order of magnitude difference in CO_2_ levels (43, 19, 9, 3 μmol kg^−1^, respectively) based on DIC speciation calculations (Table S1). Before experiments were run, the seawater O_2_ content was reduced to ~80% saturation by bubbling with N_2_ gas to ensure O_2_ did not reach super-saturation during incubations. The seawater O_2_ levels were approximately 200–300 μmol L^−1^ during the incubations (e.g., Fig. S1) within the range of 100% O_2_ solubility at 27 °C and 36 psu salinity (203 μmol L^−1^).

Photosynthesis-irradiance (PI) curves were determined using an O_2_ electrode and data acquisition system which recorded O_2_ concentrations every second (Chlorolab 3 System, Hansatech Instruments Inc.). The O_2_ electrode was calibrated daily. Light was provided by an LED light source (LH36/2R, Hansatech, UK), calibrated daily with a 2π PAR quantum sensor (LI-190, LI-COR Inc.) held up to the chamber’s glass portal, and subsequently checked at 3 light levels (50, 500, 1000 μmol photon m^−2^ s^−1^) with a resulting accuracy of approximately ±5 μmol photon m^−2^ s^−1^. The Chlorolab 3 was programmed to increase light every two minutes to preset irradiances (0, 50, 100, 200, 400, 600, 900, 1200 μmol photon m^−2^ s^−1^); this resulted in 16 min incubations. A short incubation time of 16 minutes resulted in minimal changes of seawater pH (average ±0.01) during each incubation. The 120 points over two minutes at each light level were linearized and the slopes used to calculate the rate of O_2_ flux (Fig. S1). Irradiance values covered the range measured at the bottom (~3 m) of the collection site (~600–1000 μmol photon m^−2^ s^−1^). In the Chlorolab 3 system, the light source is projected from one side of the chamber, thus the respiration:photosynthesis ratio in this system would be expected to be lower than field conditions, resulting in relatively high compensating irradiances; however, all algae were subjected to the same chamber conditions across treatments. Each algal sample was dark acclimated for ~5 minutes prior to experimentation. Water temperature was controlled using a circulating water bath set to 27 °C. Each replicate (n = 6–8) of 0.5 g fresh tissue mass of calcified species or 0.25 g fresh tissue mass of fleshy species was placed into the 20 mL Chlorolab chamber with filtered (0.45 μm) seawater. O_2_ flux rates were normalized to fresh tissue mass with the exception of CCA, which was normalized to surface area. PI curves were calculated using a hyperbolic regression model (^39^, P_net_ = P_max_ × tanh (αI/P_max_) + R) and photosynthetic parameters calculated using Excel’s data solver tool^40^. Parameters included photosynthetic efficiency (α), maximum net photosynthesis (P_max_), maximum gross photosynthesis (P_gmax_), light compensation point (I_c_), and respiration (R).

### Inhibitor Experiments

Photosynthetic rates were determined in the presence and absence of inhibitors that blocked specific inorganic C uptake mechanisms. Each experimental run used different individuals resulting in ~320 total runs (8 sp × 8 replicates × 5 [1 control and 4 inhibitors]). Inhibitors were chosen based on previous studies which identified inorganic carbon uptake mechanisms in algae^23,25,26^. Inhibitors included acetazolamide (AZ, Sigma Aldrich) that blocks the dehydration of HCO_3_^−^ into CO_2_ via external carbonate anhydrase (CA_ext_)^26^, pyridoxal (5) phosphate (PLP, Fisher Scientific) that inhibits active uptake of HCO_3_^−^, Tris buffer (Trizma R, Sigma Aldrich) that interferes with proton pump acidification of the thalli boundary layer^25^ and sodium orthovanadate (vanadate, Sigma Aldrich) that obstructs plasmalemma ATPase H^+^ pumps^23^. Solutions of AZ (200 μM), PLP (480 μM) and Tris (50 mM) were dissolved in filtered seawater (0.45 μm) followed by pH adjustment to 8.1 (1 M HCl). A 200 mM stock solution of vanadate was prepared by dissolving sodium orthovanadate in deionized water and activated using several cycles of boiling, cooling, and adjusting the pH to 10. For each experimental replicate, vanadate stock solution was added to filtered seawater for a final concentration of 400 μM. The seawater pH was checked after adding the vanadate stock solution. Concentrations of each inhibitor were chosen based on previous studies^25,26,41^ and preliminary dose response curves.

Photosynthetic rates were measured by O_2_ evolution in the same Chlorolab 3 system as used in the pH experiments. Irradiance levels, incubation time, normalization to fresh tissue mass or surface area, PI curve construction and parameter determination were also as described above for pH experiments.

### pH and AZ Interaction Experiments

To determine the effects of pH on CA_ext_-supported photosynthesis, PI curves were established across a range of pH in the presence and absence of AZ. Two species, *C*. *cervicornis* and *J*. *adhaerens*, were chosen for these experiments based on a significant CA_ext_ and pH response in preliminary studies. Photosynthetic rates were measured by O_2_ evolution as in the pH and inhibitor experiments.

### δ^13^C Isotope Analysis

Fresh tissue samples (n = 5) of each species were collected at the Looe Key patch reef site for δ^13^C isotope analyses. Upon returning to the lab, tissues were acidified to remove carbonates, dried at 60 °C to constant weight, and ground with a mortar and pestle for analysis. Tissue δ^13^C was determined using a mass spectrometer (Thermo Electron DeltaV Advantage) coupled with a CNS Elemental Analyzer (ConFlo II interface linked to a Carlo Erba NA 1500) at the Stable Isotope Mass Spec Lab (Geosciences, University of Florida). All carbon isotope results are expressed in standard delta notation relative to VPDB.

### Statistical analyses

Statistical tests (ANOVA and regression analyses) were conducted using R^42^ and SigmaPlot (v13 Syststat Software Inc.). Assumptions of normality of residuals and homogeneity of variance were examined using a Shapiro Wilkes and Levene’s test, respectively. For parameters where assumptions were not met, data was transformed with a log or square root transformation. In the case that homogeneity of variance was not met after transformations, a non-parametric Kruskal-Wallis rank sum test was used in place of an ANOVA. The effects of pH treatments and inhibitors on PI parameters (α, P_max_, P_gmax_, I_c_, and R) were tested using ANOVA. A post hoc Tukey pairwise comparison test was used to determine which pH levels had significant differences in photosynthetic parameters. A post hoc Dunnett test was used for the inhibitor experiment to compare each inhibitor group to the control. For the pH x AZ experiment significant P_gmax_ was determined for *C*. *cervicornis* and *J*. *adhaerens* using a two-way ANOVA followed by a post hoc Tukey pairwise comparison test. Data was transformed using a square root transformation. Differences in species δ^13^C signatures were determined using a Kruskal Wallis test on ranks followed by Dunn’s test. ANOVA tables for pH and inhibitor experiments are presented in the supplement.

### Data availability

Examples of raw data generated from this study are graphed in the supplement, including an example of the linearity of O_2_ flux used to calculate photosynthetic rates at each light level and the average results from pH and inhibitor experiments as a function of irradiance. Statistical tables and average values generated from the study presented in graphs are also included in the supplement. All other datasets are available from the corresponding author upon request.

## Results

### pH Experiments

**Figure 1:**
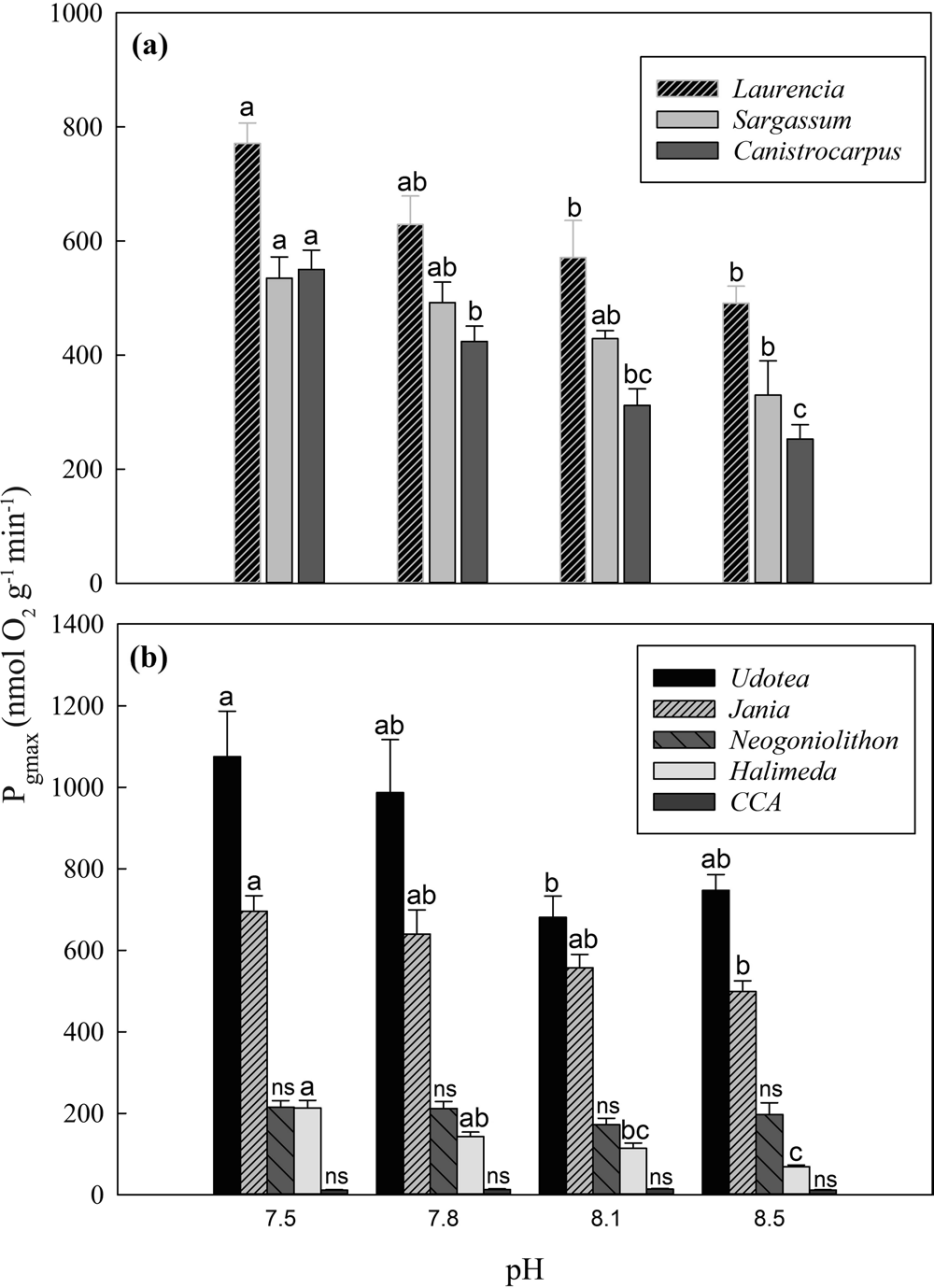
Maximum gross photosynthetic rates (P_gmax_) for fleshy (**a**) and calcified (**b**) tropical macroalgae from the Florida Reef tract across a range of pH based on PI curves (Fig. S2). Different letters signify significant differences (P < 0.05) among pH treatments within species (n = 6–8). ns = non-significant.

The greatest effect of lower pH and higher *p*CO_2_ on tropical macroalgal photosynthesis was on maximum photosynthetic rates, as illustrated by the significantly higher P_gmax_ (Fig. 1) parameter calculated from PI curves (Fig. S2 and Tables S2 and S3). There were significant effects of pH on P_gmax_ for fleshy (Fig. 1a; *L*. *intricata*, *S*. *fluitans*, and *C*. *cervicornis*) and calcified algal (Fig. 1b; *J*. *adhaerens*, *H*. *opuntia*, *U*. *luna*) species. While comparison among mean P_gmax_ was primarily significant between pH 7.5 and 8.5 using ANOVA, regression analysis indicated significant trends across the four pH treatments. There was a significant linear increase in P_gmax_ from pH 8.5 to 7.5 for all fleshy (slopes 207, 299, 269; R^2^ ≥ 0.95; *S*. *fluitans*, *C*. *cervicornis*, *L*. *intricata*) and two calcified species (slopes 138, 203; R^2^ ≥ 0.96; *H*. *opuntia*, *J*. *adhaerens*) that exhibited pH end member differences in P_gmax_, suggesting that the trend in elevated photosynthesis has the potential to track pH declines with OA. Two red calcifiers showed no significant trends (slopes <0.02; R^2^ = 0.02; *N*. *strictum*, CCA) using regression analysis across the four pH levels and no significant differences among means based on ANOVA.

All species with significant pH effects on P_gmax_ also showed similar pH effects on P_max_ with the exception of *S*. *fluitans*. *Sargassum fluitans* respiration rates were lower at 8.5 compared to 7.8 pH, although were similar (239, 271, 209, 166 nmol O_2_ g^−1^ min^−1^) across pH treatments (7.5, 7.8, 8.1, 8.5) and without a linear trend (Tables S2 and S3). *Laurencia intricata*, *S*. *fluitans* and *U*. *luna* also had higher photosynthetic efficiencies (α) at lower pH (p < 0.05, Table S2) with *H*. *opuntia* approaching significance (p = 0.07). In the two fleshy species, *L*. *intricata* and *S*. *fluitans*, α significantly increased as a function of decreasing pH (R^2^ = 0.99 and 0.88, respectively). *Halimeda opuntia*, *L*. *intricata*, and *U*. *luna* also had lower irradiance compensation points at lower pH (Table S2).

**Figure 2:**
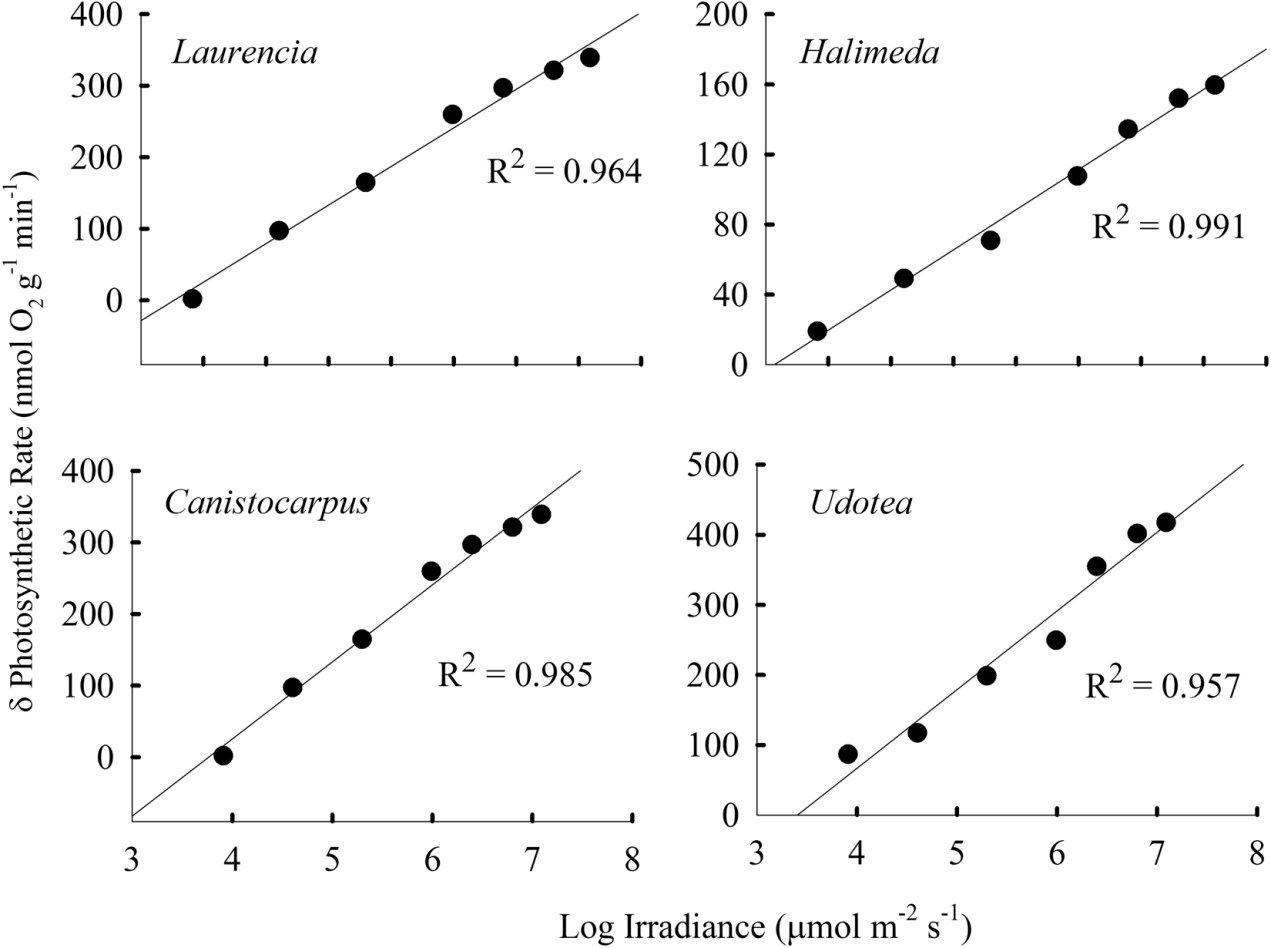
Linear relationship between the log of irradiance (μmol m^−2^ s^−1^) and the difference in photosynthetic rates at 8.5 and 7.5 pH for two species of fleshy (left panels) and calcified (right panels) tropical macroalgae with greater P_gmax_ under OA conditions (Fig. 1). Data from PI curves shown in Fig. S2.

The PI curves showed the difference in photosynthetic rates between pH treatments increased with irradiance (Fig. S2) in the six species that showed a significant P_gmax_ or P_max_ response to pH treatments (Fig. 1 and Table S3). Comparing the differences (δ) in photosynthetic rates of the two pH end members (8.5 and 7.5) across all light levels produced a linear relationship between δ photosynthetic rate and the log of irradiance in two green calcifying (*H*. *opuntia*, *U*. *luna*) and two fleshy (*L*. *intricata*, *C*. *cervicornis*) species (Fig. 2). The calcifying species, *J*. *adhaerens*, exhibited a linear relationship between photosynthetic rates between the two pH end members and irradiance (slope = 0.15; R^2^ = 0.98), and the fleshy species, *S*. *fluitans*, showed linearity at low irradiance (slope = 0.13; R^2^ = 0.91; up to 200 μmol photon m^−2^ sec^−1^) and then a modest decline above saturation (slope = −0.08; R^2^ = 0.99; ≥600 μmol photon m^−2^ sec^−1^). These data indicate that irradiance is critical to the enhanced photosynthetic response under OA conditions in both fleshy and calcified macroalgae that respond to elevated *p*CO_2_.

### Inhibitor experiments

**Figure 3:**
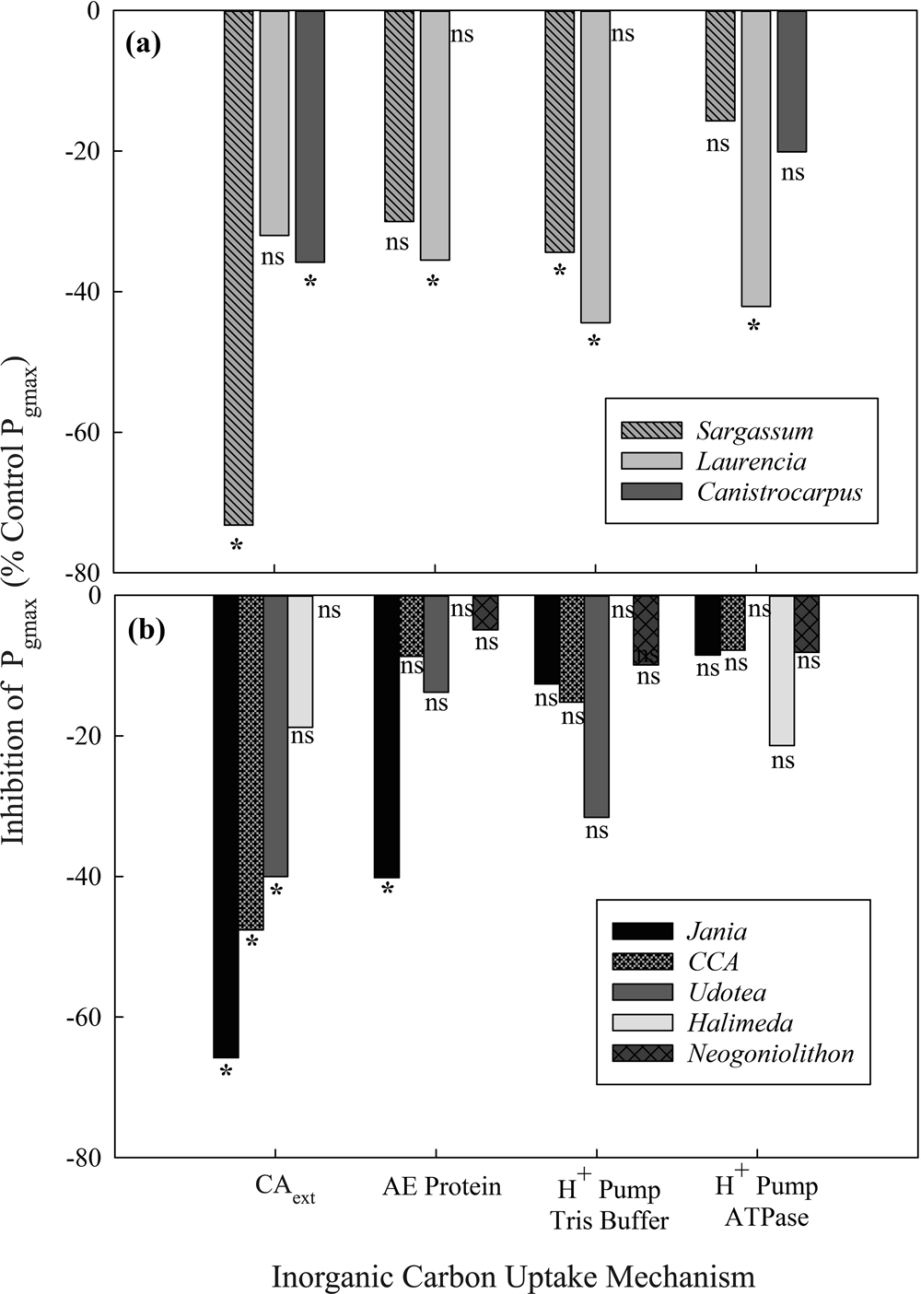
Inhibition of P_gmax_ in three fleshy (**a**) and five calcifying (**b**) tropical macroalgae as inhibitor block various bicarbonate uptake pathways: external carbonic anhydrase (CA_ext_) with acetazolamide (AZ), AE protein by pyridoxal (5) phosphate (PLP), proton pump acidification by Tris buffer and ATPase H^+^ pumps by sodium orthovanadate.

Two of the three fleshy species P_gmax_ and/or P_max_ rates were significantly lowered with the inhibition of CA_ext_ by AZ (Fig. 3a and Tables S5 and S5) based on PI curves (Fig. S3). *Laurencia intricata* was the only fleshy species that indicated a dependency of P_max_ and P_gmax_ on several other bicarbonate use mechanisms, including AE protein driven active HCO_3_^−^ uptake and H^+^ pumps, potentially an ATPase, as indicated by blockage with vanadate (Fig. 3a). However, the PLP and Tris buffer also raised the respiration rates of *L*. *intricata*. *S*. *fluitans* may utilize H^+^ pumps, based on Tris buffer inhibition, but no significant ATPase-linked pump was detected, suggesting other H^+^ pumps may be functioning. Calcifying species were also primarily dependent on CA_ext_ for HCO_3_^−^ acquisition with four of the five species showing significant declines (40–65%) in P_gmax_ and/or P_max_ (Tables S4 and 5) in the presence of AZ (Fig. 3b). *Jania adhaerens* was the only calcified species that indicated the potential use of active HCO_3_^−^ uptake and no calcifiers appeared to utilize proton pumps based on the inhibitor PLP. Curiously, *Neogoniolithon strictum*, did not elicit a significant photosynthetic response to any of the inhibitors (Figs 3b and S3).

### pH and AZ Interaction Experiments

**Figure 4:**
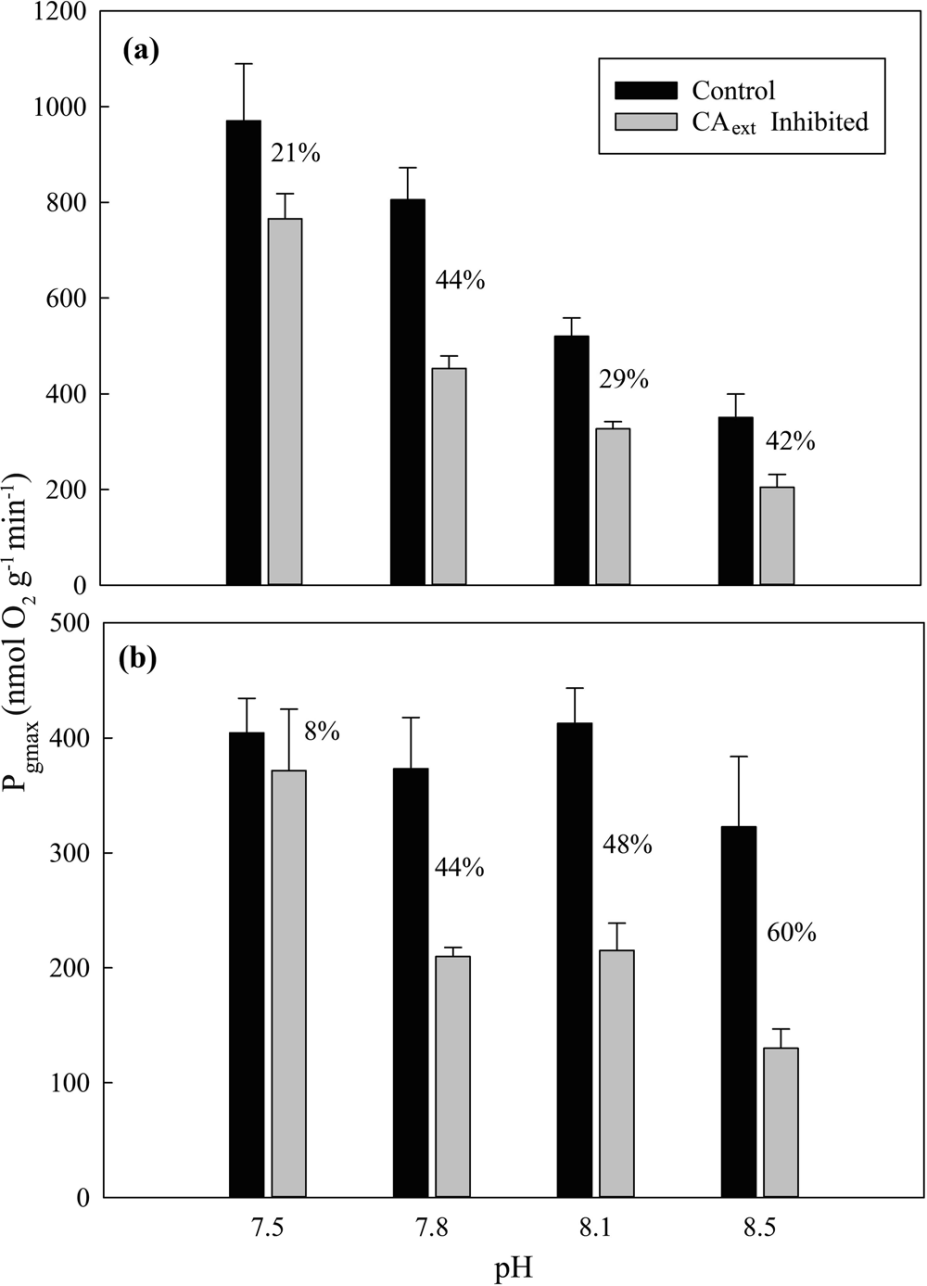
The effect of external carbonic anhydrase (CA_ext_) inhibition by acetazolamide (AZ) on maximum photosynthetic rates (P_gmax_) for (**a**) *Canistrocarpus cervicornis* and (**b**) *Jania adhaerens* at four pH levels. Data are means with SE (n = 7–8). P_gmax_ was calculated from PI curves fit to a hyperbolic tangent equation^39^. Percent differences between control and CA_ext_ inhibited P_gmax_ rates are shown.

The relative importance of CA_ext_, the major HCO_3_^−^ use pathway identified for the majority of species examined in this study (Fig. 3), was lowest (8–21%) at pH 7.5 (Fig. 4). Significant pH and AZ inhibitor effects were found in both species (P < 0.01, Fig. 4). P_gmax_ increased in response to lower pH for *C*. *cervicornis* in controls between all pH levels, while differences were found between all pH levels except 7.8 and 8.1 in the presence of AZ. Within all pH levels, *C*. *cervicornis* P_gmax_ was higher in controls than with AZ except at pH 7.5. In contrast to *C*. *cervicornis*, *J. adhaerens* P_gmax_ was significantly higher at pH 7.5 with AZ. Regression analysis of P_gmax_ as a function of pH treatment levels supported the 2-way ANOVA results, showing significant declines in maximum P_gmax_ for both species with AZ, but only for *C*. *cervicornis* controls. *C*. *cervicornis* photosynthetic rates declined similarly with increasing pH with and without CA_ext_ (slope = −528 and −643, respectively; p < 0.01), with an approximately 30% decline without CA_ext_. In contrast, *J*. *adhaerens* showed no significant decline in photosynthesis with CA_ext_ (slope = −66; p = 0.07) as pH increased, compared to a decline without CA_ext_ (slope = −220; p < 0.01). These data, together with those in Fig. 3, support the idea that the dependency of tropical marine macroalgae on HCO_3_^−^, and specifically CA_ext_, to sequester DIC may decline as OA provides greater CO_2_ availability under a lower pH.

### δ^13^C Isotope Analysis

**Figure 5:**
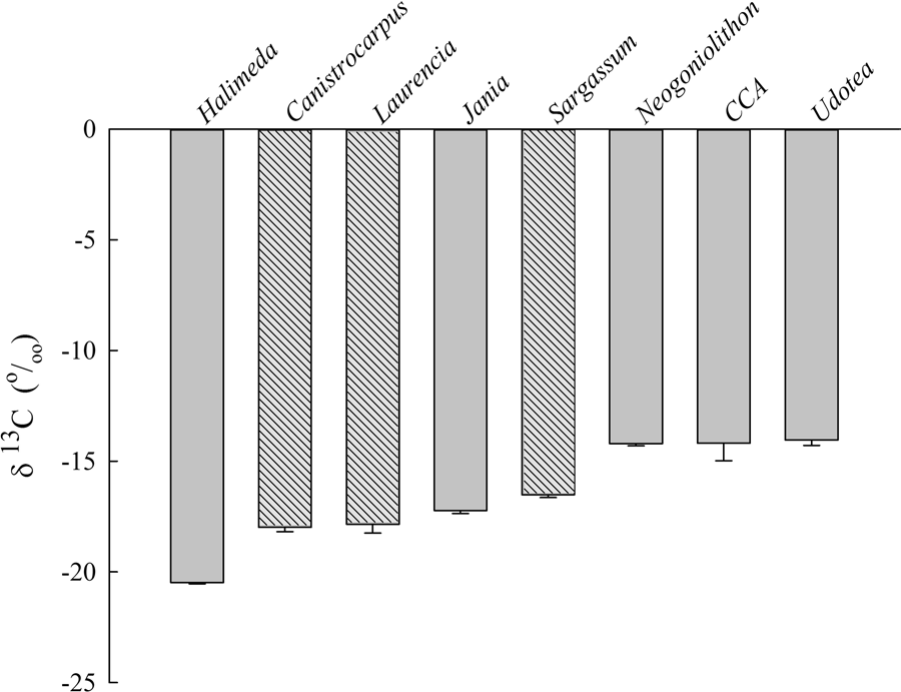
Organic tissue δ^13^C isotope signatures of calcifying (grey; *Halimeda opuntia*, *Jania adhaerens*, *Neogoniolithon strictum*, CCA, *Udotea luna*) and fleshy (striped; *Canistrocarpus cervicornis*, *Laurencia intricata*, *Sargassum fluitans*) macroalgae used in this study collected from a patch reef site on the Florida Keys reef tract.

The organic tissue δ^13^C signatures for all species examined fell within a relatively narrow range between −14 and −20 (Fig. 5). Statistical differences among species was significant based on a Kruskal-Wallis test (df = 7, Χ^2^ = 34.4, p = 1.3E-5) with *H*. *opuntia* exhibiting a more depleted δ^13^C signature than *S*. *fluitans*, *N*. *strictum*, CCA and *U*. *luna*. There were also significant differences between *C*. *cervicornis* and *U*. *luna*. Further, the two red species (*J*. *adhaerens*, *L*. *intricata*) that responded significantly to pH treatments had 20% lighter signatures than the two red species that did not (*N*. *strictum*, CCA).

## Discussion

Although the tropical macroalgal species examined showed evidence of HCO_3_^−^ use, all the fleshy (100%) and a high proportion of the calcified (40%) species had the ability to increase photosynthesis under OA conditions, especially at high irradiance. Our results support the supposition that inorganic carbon physiology may underlie macroalgal responses to elevated *p*CO_2_. These data concur with those from field studies at naturally high CO_2_ sites where flexibility to utilize CO_2_ for photosynthesis increased a species abundance in the community adjacent to the CO_2_ seeps^18^. Several experimental and culture studies have also shown an increase in photosynthesis and growth in temperate macroalgae in response to elevated *p*CO_2_^43,44^. Others suggest that greater CO_2_ availability only advantages macroalgae that solely depend on CO_2_ diffusion and lack CCMs^7,30,45,46^. We suggest that facultative use of HCO_3_^−^ under greater access to CO_2_, regardless of CCM mechanism employed, is more likely to predict tropical macroalgal responses to OA, particularly if they are not light limited.

Given that most macroalgae reside primarily on the benthos where the light environment can vary substantially, understanding the interactive effects of elevated *p*CO_2_ and irradiance is critical to predict how macroalgae are likely to respond to OA. Six of the eight species studied, including both fleshy and calcifying species, increased maximum photosynthetic rates at light saturation (P_gmax_ or P_max_) as a result of lower pH and higher *p*CO_2_, indicating the importance of irradiance for the OA response in these autotrophs. Irradiance influenced the degree to which species responded to lower pH. Maximum photosynthetic rates increased as a log or linear function of irradiance in six species under OA conditions and the photosynthetic efficiency parameter (α) significantly increased in two species as a function of decreasing pH. Thus, the specific photosynthetic response to changes in pH was dependent on the light gradient. Since all species within the patch reef community sampled were growing in a high-light environment (700–1200 μmol photons m^−2^ s^−1^), light limitation should not constrain their response to elevated *p*CO_2_ and their ability to utilize CCMs in the patch reefs of the Florida Reef Tract. However, under lower light, macroalgae may be saturated with respect to inorganic carbon, where light is the primary limiting factor^47^, or CCMs may be downregulated due to low-energy availability. Based on a survey of macroalgae on the Great Barrier Reef, the only non-CCM species were from deep (10 m) reef sites^9^, consistent with the findings of non-CCM macroalgal species from deep temperate rock reefs^32,45^. Thus, CCMs likely require adequate irradiance to support energy-dependent C-uptake of HCO_3_^−^, but the carbon demand under high irradiance also increases and is likely satiated by greater CO_2_ uptake when available.

Interestingly, the three non-CCM species identified by δ^13^C ratios <−30‰ found at depth on the Great Barrier Reef ^9^ were fleshy red species, while the calcifying red and green species had stable isotope ratios (−13 to −19‰) indicative of both HCO_3_^−^ and CO_2_ use, consistent with those found on patch reefs in this study and the majority (~75% or more) of macroalgae on the Great Barrier Reef. While the literature to date indicate a lower photosynthetic response to OA by calcifying versus fleshy algae in temperate macroalgae^8^, our results with tropical calcifying species present a diversity of HCO_3_^−^ use mechanisms and CO_2_ uptake responses. For example, pH had no effect on photosynthesis in the red calcareous species *N*. *strictum* and the CCA assemblage. These two species also presented the highest δ^13^C signatures amongst the eight species, suggesting more dependency on HCO_3_^−^ and/or less ability to sequester CO_2_. However, based on inhibitor studies, no HCO_3_^−^ uptake mechanism was discerned for *N*. *strictum*. In contrast, the red calcareous alga, *J*. *adhaerens*, showed a statistically significant difference in P_max_ and P_gmax_ as a function of pH and had a moderately lower δ^13^C signature than *N*. *strictum* and CCA. These data confirm that, while some red calcareous algae lack a photosynthetic OA response^8,48,49^, there are exceptions^13,50^. Our results support the contention that ecologically important red calcifiers should be examined for their species-specific OA responses. This is particularly important in the red phyla because of its bimodal distribution in δ^13^C signatures (peak at −35 and −20) compared to green and brown phyla with a unimodal peak (peak at −20) based on a global meta-analysis of marine macroalgae^51^. In the two green calcifying species, *H*. *opuntia* and *U*. *luna*, photosynthetic rates significantly increased at low pH and were highly correlated to the log of irradiance. The results for *H*. *opuntia* are in contrast with others that found no or negative effects of *p*CO_2_ on photosynthesis^8,11,52–54^, but isotope signatures from this study, and those from the Great Barrier Reef^9^, indicate both HCO_3_^−^ and CO_2_ uptake. Calcifiers are also unique in that CO_2_ can become more available via the calcification process, thus more research is needed to understand linkages between photosynthesis and calcification.

In contrast to calcifiers, all three-fleshy species studied, including those from brown, *C*. *cervicornis*, *S*. *fluitans*, and red, *L*. *intricata*, phyla increased P_gmax_ and/or P_max_ at lower pH. This is consistent with a high percentage of species within the brown phyla show increases in photosynthesis under OA conditions^29,55–57^ and fleshy species in general increasing percent coverage near natural CO_2_ seeps^35,58^ at the expense of calcifiers. The idea that calcifiers do not respond photosynthetically as a group to elevated *p*CO_2_ may be confounded by observations that they decline in the field adjacent to CO_2_ seeps. This observation may be better explained by either direct OA effects on their carbonate thalli structure, due to changes in calcification and dissolution processes, or dominance by fleshy species that are more successful competitors for resources under OA conditions^59^.

Even though the majority of calcifiers and all the fleshy species’ photosynthetic rates increased in response to elevated *p*CO_2_, they all showed evidence of HCO_3_^−^ use based on stable isotope ratios. The δ^13^C range between −14 and −20 imply HCO_3_^−^ use in photosynthesis for the tropical macroalgae studied. The most prevalent mechanism for HCO_3_^−^ uptake was the use of CA_ext_ shown by the reduction in photosynthesis in the presence of AZ. Two fleshy and one calcifying species also showed evidence for active uptake and proton pumping to facilitate HCO_3_^−^ exchange. Evidence for an AE protein role was found for the first time in *J*. *adhaerens* and *L*. *intricata*. To date, the evidence for AE proteins has only been found in temperate brown^29^ (*Macrocystis pyrifera*), green and red species^26^. Acidification of the boundary layer was important for *S*. *fluitans* and *L*. *intricata* based on Tris inhibition. One mechanism for acidification of the boundary layer is the use of an active ATPase H^+^ pump, but only *L*. *intricata* showed evidence for proton pumping in the inhibitor experiments. Based on inhibitor studies, it appears that several mechanisms exist in tropical macroalgae to moderate CO_2_ limitation at current seawater CO_2_ concentrations by gaining access to the more abundant HCO_3_^−^ ion. A meta-analysis of 613 species world-wide showed δ^13^C values increase with decreasing latitude for both brown and red phyla, indicating the significance of CCMs in tropical macroalgal species^51^. However, δ^13^C values still ranged between −10 and −30 in these two phyla globally, demonstrating the diversity of C-uptake strategies in tropical macroalgae.

As CO_2_ becomes more available to HCO_3_^−^ users under low pH, the dependency on HCO_3_^−^ use via CA_ext_ appears to be dampened, as was shown for *C*. *cervicornis* and *J*. *adhaerens*. This finding is consistent with recent volcanic CO_2_ seep studies that found lowered δ^13^C values in macroalgae closest to natural CO_2_ seeps^18^. At this site, HCO_3_^−^ users were hypothesized to have decreased HCO_3_^−^ uptake and acquired more CO_2_. The greater CO_2_ use by macroalage under long-term elevated CO_2_ exposure at vents support our conclusion, using highly controlled short-term experiments, that macroalgae utilizing HCO_3_^−^, and constrained by low *p*CO_2_ in seawater, are likely to take advantage of the greater CO_2_ availability as the oceans become more acidic. Because marine macroalgae developed HCO_3_^–^ use mechanisms to facilitate CO_2_ acquisition in seawater with low CO_2_ availability, it is reasonable to assume they would take advantage of increased seawater *p*CO_2_, supported by trends in greater CO_2_ use in macroalgae with increasing latitude^51^. In the field, CO_2_ availability can be enhanced from short diurnal cycles of community metabolism where respiration rates exceed photosynthesis and from longer trends controlled by cultural eutrophication and OA. The potential for some fleshy macroalgal species to decrease HCO_3_^−^ uptake or increase gross photosynthetic production with greater CO_2_ availability can result in elevated growth and abundance, as has been shown experimentally^8,14^ and reported from naturally acidified volcanic seeps^17,18^.

We conclude that tropical macroalgae that utilize multiple mechanisms for HCO_3_^−^ uptake can increase their photosynthetic rates under low pH and greater CO_2_ availability. The consistent positive photosynthetic response to OA by fleshy species, in contrast to a split amongst calcifiers, support the possibility that fleshy forms could outcompete calcifiers. While we present data that tropical macroalgae utilize a diverse suite of HCO_3_^−^ use pathways, some species are likely to become less dependent on HCO_3_^−^ as an inorganic C source for photosynthesis under OA. This study also clearly indicates the importance of irradiance in controlling the photosynthetic response of tropical marine macroalgae to OA. Our results show that at low irradiance, light limitation, rather than carbon limitation, probably controls photosynthetic responses to OA. This presents two important implications for OA research: (1) studies must take careful measurements and control light levels in order to compare amongst OA studies, (2) in low-light environments, such as on deep reefs and under ledges, elevated *p*CO_2_ and a lower pH may not affect photosynthesis of macroalgae unless CO_2_ is limiting and HCO_3_^−^ use is energetically constrained, and (3) the major shifts in photosynthetically-driven macroalgal changes due to OA will most likely occur in moderate to high-irradiance environments when CCMs are inefficient at meeting the C-demands of photosynthesis and greater access to CO_2_ enhances direct and/or indirect competitive interactions. While we identify diverse C-use strategies for photosynthesis in tropical macroalgae using inhibitors, this approach is complicated by species-specific responses due to thalli structure and potential effects on non-target metabolic functions, thus more work is needed to further support C-use mechanisms presented herein. Further, studies that identify the importance of elevated photosynthesis to growth for these species are warranted.

## Electronic supplementary material

Supplementary Information
