# Supplementary material for: The role of irradiance and C-use strategies in tropical macroalgae photosynthetic response to ocean acidification
Source: Sci Rep. 2018 Jun 21;8:9479. doi: 10.1038/s41598-018-27333-0 (PMC6013460; doi:10.1038/s41598-018-27333-0)
Supplement: Supplementary file 1 — Supplementary Information [file 41598_2018_27333_MOESM1_ESM.pdf]

The role of irradiance and C-use strategies in tropical macroalgae photosynthetic response to ocean acidification

Regina C Zweng[1], Marguerite S Koch[1,*], George Bowes[2]

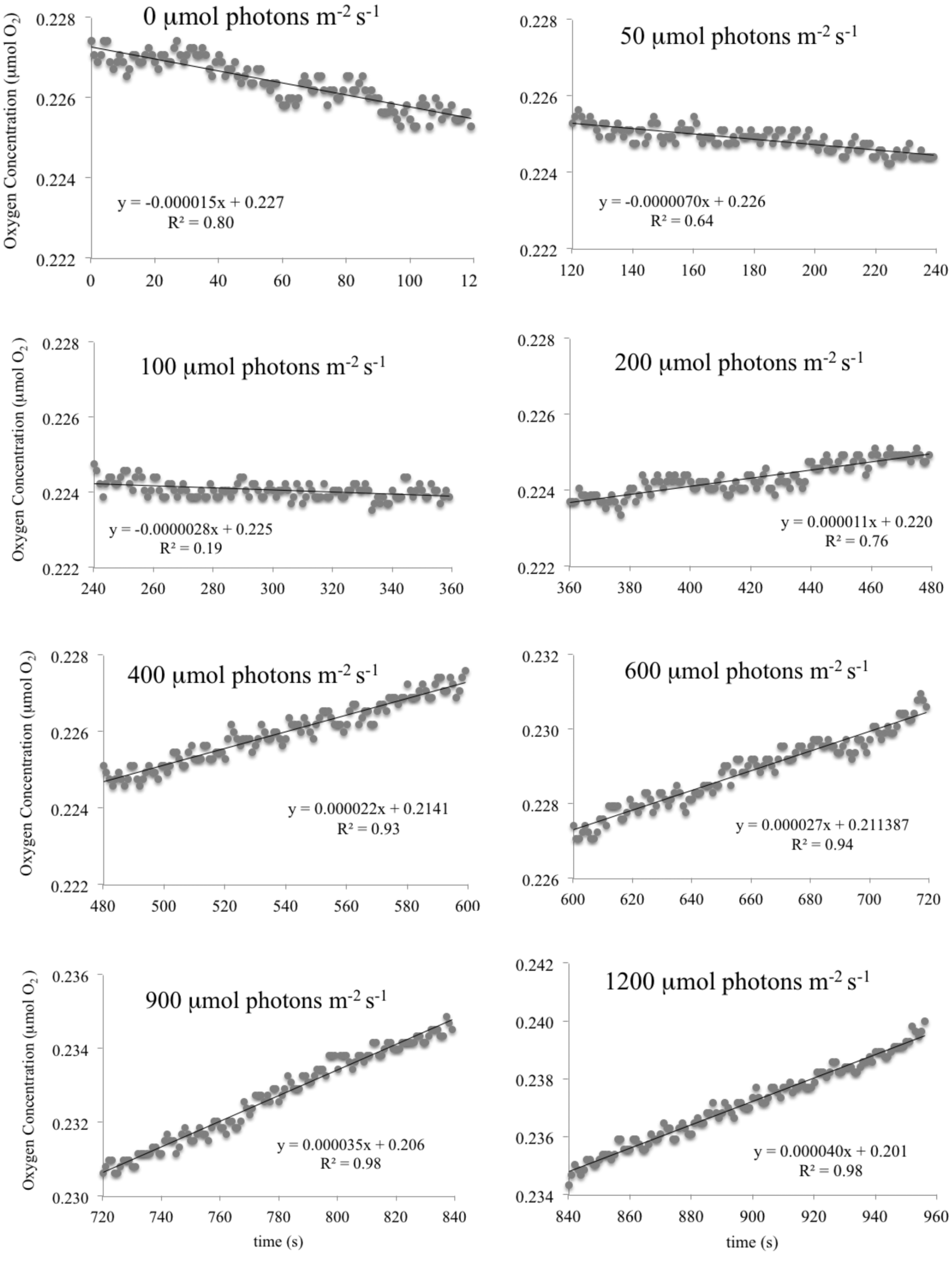

Figure S1. Raw data run illustrating linearity after 2 minute incubations using the highly sensitive Chlorolab 3 system providing O_2 concentration (μmol mL^{-1}) readings every second. The eight panels show raw data for *Laurencia* at ambient pH across the eight-irradiance levels examined. If linearity was not achieved, a new sample was measured, but the majority of runs produced similar raw data files from which PI curves were established. Each replicate PI curve for each treatment was conducted using a new individual.

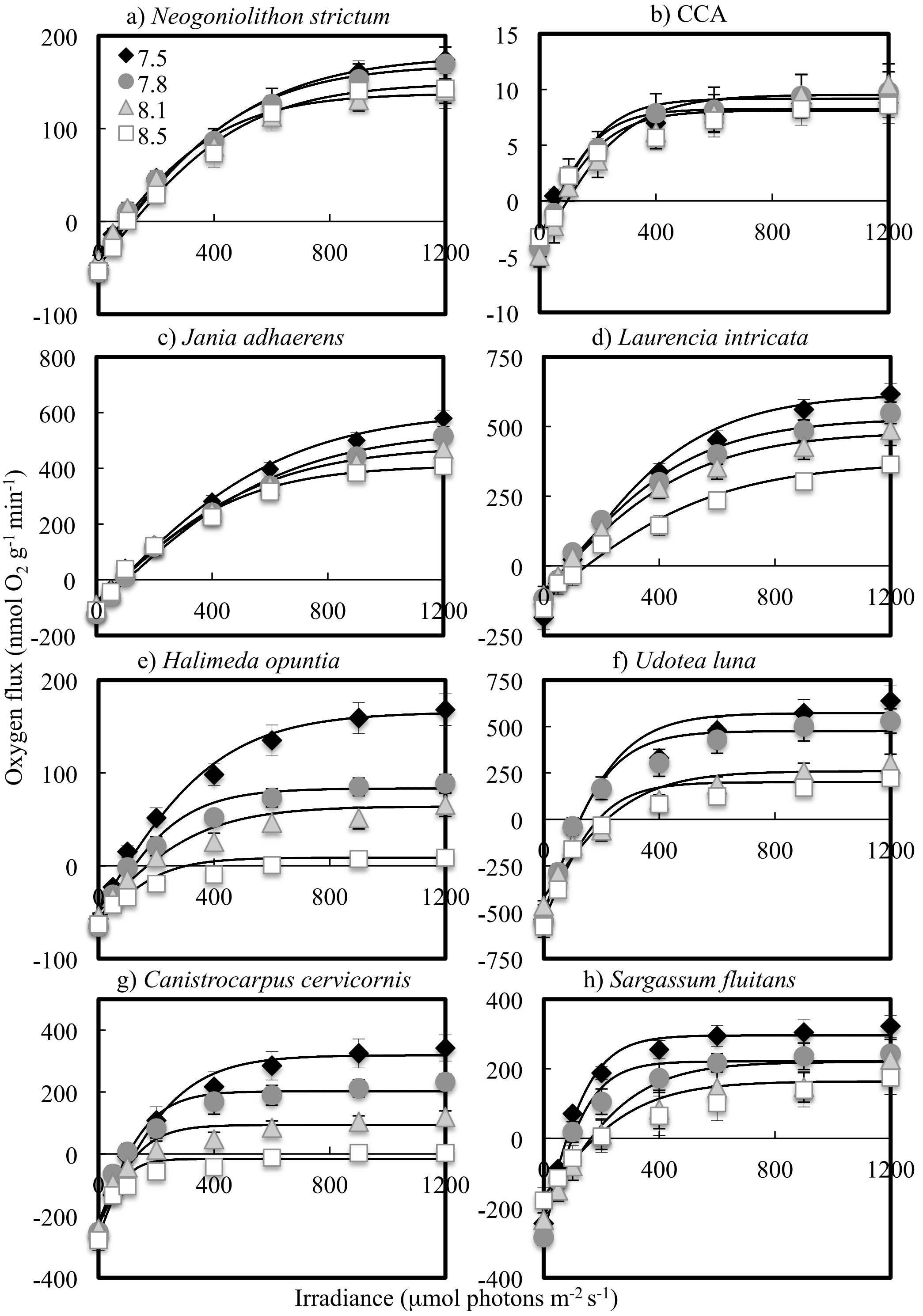

Figure S2. Photosynthesis–Irradiance (P:I) curves for all species at the four pH levels examined (means ± SE of 6-8). P:I curves were fit to a hyperbolic tangent curve (Jassby and Platt 1976) from which photosynthetic parameters were calculated (see Table S3).

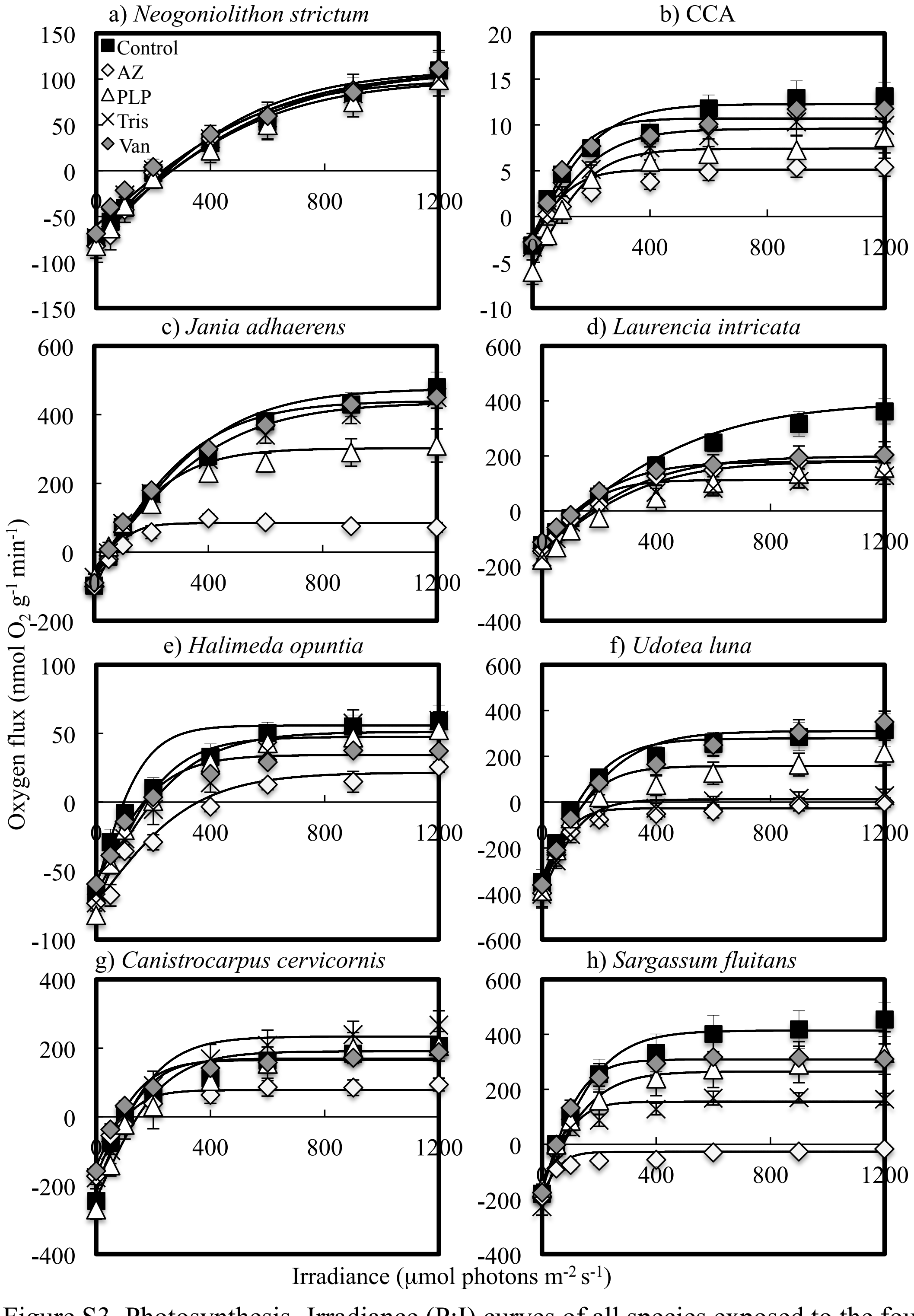

Figure S3. Photosynthesis−Irradiance (P:I) curves of all species exposed to the four inhibitors (see text details): AZ (white diamond), PLP (white triangle), Van (gray diamond), and Tris (black x); and a control (black square). Data are means with SE (n = 8). P:I curves were fit to a hyperbolic tangent curve (Jassby and Platt 1976) from which photosynthetic parameters were calculated (See Table S5).

Table S1 Inorganic carbon (µmol kg^{-1}) and CO_2 in ppm (to compare with atmospheric concentrations) based on pH treatments and speciation calculations (CO2sys, Pierrot et al., 2006) using average in situ salinity (37 psu), incubation temperature (27°C) and measured alkalinity (see methods).

pH Treatment	CO_2 (ppm)	CO_2	HCO_3^- (µmol kg^{-1})	CO_3^{2-}
7.5	1645	43.4	2161	85
7.8	741	19.4	1984	161
8.1	334	9.0	1774	277
8.5	127	3.4	1531	540

Table S2. One-Way ANOVA results on net photosynthesis maxima (P_{max}), gross photosynthesis maxima (P_{gmax}), photosynthetic efficiency (α), respiration (R) and light compensation point (I_c) in response to pH treatments. Significant effects ($p < 0.05$) are marked with an asterisk.

	df	Sum Sq	Mean Sq	F-value	p-value
Canistrocarpus cervicornis					
P_{gmax}	3	0.25791	0.08597	20.65	9.52e-06*
P_{max}	3	0.31076	0.10359	25.37	2.54e-06*
R	3	0.00815	0.002716	1.073	0.388
α	3	1.617e-06	5.391e-07	0.338	0.798
I_c	3	13575	4525	0.51	0.682
CCA					
P_{gmax}	3	0.0000391	1.302e-05	0.676	0.574
P_{max}	3	0.0000116	3.856e-06	0.208	0.89
R	3	0.000455	0.0001517	0.748	0.533
α	3	1.228e-09	4.093e-10	0.535	0.662
I_c	3	0.1504	0.05014	0.519	0.673
Halimeda opuntia					
P_{gmax}	3	0.16070	0.05357	23.18	9.59e-08*
P_{max}	3	0.3347	0.11157	35.73	1.04e-09*
R	3	0.001075	0.0003583	2.073	0.126
α	3	3.181e-07	1.061e-07	2.586	0.073 .
I_c	3	1.6296	0.5432	16.5	2.73e-06*
Jania adhaerens					
P_{gmax}	3	0.1824	0.06079	4.531	0.0104*
P_{max}	3	0.08057	0.026858	4.156	0.0148*
R	3	0.00266	0.0008882	0.681	0.571
α	3	5.660e-08	1.886e-08	0.317	0.813
I_c	3	6717	2239	1.035	0.392
Laurencia intricata					
P_{gmax}	3			9.6251	0.02204[i]*
P_{max}	3	0.2058	0.06861	5.465	0.00551*
R	3	0.01580	0.005267	1.499	0.241
α	3	1.869e-06	6.229e-07	3.279	0.0392*
I_c	3	19102	6367	4.612	0.0114*
Neogoniolithon strictum					
P_{gmax}	3	0.00932	0.003108	0.946	0.432
P_{max}	3	0.00818	0.002727	1.161	0.342
R	3	0.001029	0.0003430	1.292	0.297
α	3	4.200e-09	1.411e-09	0.085	0.968
I_c	3	8378	2793	1.831	0.164
Sargassum fluitans					
P_{gmax}	3	0.09566	0.03189	4.908	0.0188*
P_{max}	3	0.04143	0.013810	3.241	0.0603
R	3	0.02420	0.008067	4.601	0.023*
α	3	1.502e-05	5.006e-06	4.602	0.023*
I_c	3	122441	40814	2.44	0.115

Udotea luna					
P_{gmax}	3	0.6387	0.21289	4.226	0.0182*
P_{max}	3	0.5566	0.18553	5.69	0.00551*
R	3			4.9333	0.1767[i]
α	3	0.04497	0.014990	4.288	0.0172*
I_c	3	33900	11300	4.487	0.0145*

[i]Kruskal-Wallis rank sum test

Table S3. Average (± SE) gross maximum photosynthesis (P_{gmax} nmol O_2 g^{-1} min^{-1}), net maximum photosynthesis (P_{max} nmol O_2 g^{-1} min^{-1}), respiration (R nmol O_2 g^{-1} min^{-1}) photosynthetic efficiency (α nmol O_2 / µmol photons), and light compensation point (I_c µmol photons m^{-2} s^{-1}) at pH 7.5, 7.8, 8.1 and 8.5. Photosynthetic parameters were calculated using the hyperbolic tangent equation (Jassby and Platt 1976). Different letters denote significant differences among pH treatments ($P < 0.05$). [1]Parameter values for CCA are as described above but normalized to cm^{-2}.

	P_{gmax}	P_{max}	R	α	I_c
Canistrocarpus cervicornis					
7.5	550 ± 34[a]	319 ± 42[a]	231 ± 26	1.97 ± 0.27	144 ± 40
7.8	424 ± 27[b]	203 ± 28[ab]	222 ± 10	2.39 ± 0.37	118 ± 25
8.1	321 ± 29[bc]	94 ± 17[abc]	218 ± 14	1.98 ± 0.57	190 ± 56
8.5	253 ± 25[c]	-16 ± 22[c]	269 ± 32	2.64 ± 0.86	167 ± 61
CCA[1]					
7.5	12 ± 1.4	8.2 ± 1.2	3.3 ± 0.5	0.059 ± 0.013	68 ± 10
7.8	13 ± 1.7	9.2 ± 1.7	4.0 ± 0.6	0.062 ± 0.011	107 ± 47
8.1	14 ± 1.7	9.5 ± 1.6	4.4 ± 0.9	0.048 ± 0.008	128 ± 37
8.5	11 ± 1.4	8.1 ± 1.6	3.2 ± 0.4	0.050 ± 0.004	71 ± 11
Halimeda opuntia					
7.5	213 ± 19[a]	165 ± 17[a]	48 ± 4	0.51 ± 0.06	104 ± 12[a]
7.8	143 ± 11[ab]	83 ± 9[ab]	60 ± 6	0.51 ± 0.08	144 ± 22[a]
8.1	114 ± 13[bc]	64 ± 13[bc]	50 ± 4	0.31 ± 0.07	216 ± 28
8.5	69 ± 4[c]	9 ± 4[c]	61 ± 4	0.31 ± 0.07	454 ± 67[b]
Jania adhaerens					
7.5	696 ± 38[a]	605 ± 28[a]	91 ± 14	1.07 ± 0.10	85 ± 9
7.8	640 ± 59	538 ± 64	102 ± 16	0.98 ± 10.11	119 ± 28
8.1	557 ± 33	480 ± 29	77 ± 6	0.95 ± 0.08	83 ± 7
8.5	499 ± 26[b]	410 ± 24[b]	89 ± 13	1.02 ± 0.04	90 ± 14
Laurencia intricata					
7.5	771 ± 36[a]	618 ± 38[a]	152 ± 34	1.56± 0.24[a]	99 ± 14
7.8	629 ± 50	530 ± 48	100 ± 13	1.26 ± 0.15	84 ± 12[a]
8.1	571 ± 65[b]	481 ± 55	90 ± 16	1.09 ± 0.14	83 ± 12[a]
8.5	491 ± 30[b]	371 ± 15[b]	120 ± 21	0.83 ± 0.08[b]	151 ± 20[b]
Neogoniolithon strictum					
7.5	215 ± 16	179 ± 15	36 ± 4	0.38± 0.03	97 ± 11
7.8	212 ± 17	169 ± 17	43 ± 5	0.42 ± 0.05	112 ± 16
8.1	172 ± 16	138 ± 14	34 ± 4	0.40 ± 0.03	87 ± 11
8.5	197 ± 29	149 ± 22	48 ± 9	0.40 ± 0.06	130 ± 16
Sargassum fluitans					
7.5	535 ± 37[a]	296 ± 32	239 ± 12	3.21 ± 0.21	81 ± 3
7.8	492 ± 36	221 ± 36	271 ± 25[a]	3.06 ± 0.78	114 ± 21
8.1	429 ± 14	220 ± 9	209 ± 17	1.40 ± 0.32	232 ± 101
8.5	330 ± 60[b]	166 ± 37	166 ± 26[b]	1.03 ± 0.58	297 ± 78

Udotea luna					
7.5	1075 ± 112[a]	572 ± 109[a]	503 ± 45	4.31 ± 0.93	149 ± 18[a]
7.8	987 ± 130	476 ± 66	511 ± 87	4.50 ± 1.29	148 ± 23[a]
8.1	681 ± 52[b]	260 ± 54[b]	421 ± 17	2.25 ± 0.19[a]	236 ± 24[b]
8.5	747 ± 39	200 ± 52[b]	547 ± 34	3.68 ± 0.36[b]	203 ± 15

Table S4. One-Way ANOVA table for inhibitors effects on net photosynthesis maxima (P_{max}), gross photosynthesis maxima (P_{gmax}), photosynthetic efficiency (α), respiration (R) and light compensation point (I_c). In cases where the assumption of homogeneity of variances was not met a non-parametric Kruskal-Wallis test was used (i). Significant effects ($p < 0.05$) are marked with an asterisk.

	Df	Sum Sq	Mean Sq	F-value	p-value
Canistrocarpus cervicornis					
P_{gmax}	4	0.02141	0.005352	6.32	6.49 E-4*
P_{max}	4	0.1763	0.04408	4.406	0.00562*
R	4	0.05185	0.012963	1.978	0.12
α	4	0.000182	4.549e-05	0.269	0.896
I_c	4	0.4475	0.11188	1.328	0.28
CCA					
P_{gmax}	4	0.005196	0.0012991	2.626	0.051
P_{max}	4	0.0002523	6.309e-05	3.46	0.0175*
R	4	0.004073	0.0010182	2.018	0.113
α	4	1.853e-05	4.632e-06	0.788	0.541
I_c	4	1.041	0.2601	1.913	0.13
Halimeda opuntia					
P_{gmax}	4	0.005921	0.0014804	1.599	0.198
P_{max}	4	0.00575	0.001437	2.995	0.0326*
R	4	8.170e-07	2.043e-07	1.102	0.372
α	4	0.0003989	9.972e-05	1.62	0.192
I_c	4	188376	47094	4.926	0.00343 *
Jania adhaerens					
P_{gmax}	4	0.6918	0.17295	16.95	8.1e-08*
P_{max}	4	0.8357	0.20893	19.08	2.1e-08*
R	4	0.01516	0.003790	3.635	0.014*
α	4	-	-	2.2244	0.6946[i]
I_c	4	11086	2772	2.053	0.108
Laurencia intricata					
P_{gmax}	4	0.2560	0.06400	4.652	0.0042 *
P_{max}	4	0.3773	0.09433	7.733	0.00015 *
R	4	0.04518	0.011295	3.629	0.014*
α	4	2.107e-06	5.268e-07	1.561	0.207
I_c	4	0.2866	0.07165	1.894	0.136
Neogoniolithon strictum					
P_{gmax}	4	0.00355	0.0008873	0.35	0.842
P_{max}	4	0.00163	0.0004065	0.241	0.913
R	4	-	-	3.0585	0.5481[i]
α	4	2.24e-08	5.592e-09	0.467	0.759
I_c	4	61062	15265	0.806	0.53
Sargassum fluitans					
P_{gmax}	4	0.5426	0.13565	11.72	4.95e-06*
P_{max}	4			23.2344	0.0001137[i]
R	4	0.03798	0.009494	3.023	0.031
α	4	1.438e-05	3.596e-06	1.824	0.148
I_c	3	4110	1370	1.118	0.36

Udotea luna					
P_{gmax}	4	0.03634	0.009085	3.498	0.0167*
P_{max}	4	0.7409	0.1852	12.6	1.9e-06*
R	4	0.0349	0.008735e-5	0.483	0.748
α	4	4.780e-06	1.195e-06	0.321	0.862
I_c		No hov			

Table S5. Average (± SE) gross maximum photosynthesis (P_{max} nmol O_2 g^{-1} min^{-1}), net maximum photosynthesis (P_{gmax} nmol O_2 g^{-1} min^{-1}), respiration (R nmol O_2 g^{-1} min^{-1}) photosynthetic efficiency (α µmol O_2 $nmol^{-1}$ photons), and light compensation point (I_c µmol photons m^{-2} s^{-1}) in the presence of four inhibitors (AZ, PLP, Tris, and Van) and control. Inhibitors with a significant effect ($p < 0.05$) relative to control are marked with asterisks. [1]Parameter values for CCA are as described above but normalized to cm^{-2}. Parameters were calculated using the hyperbolic tangent equation (Jassby and Platt 1976).

	P_{gmax}	P_{max}	R	α	I_c
Canistrocarpus Cervicornis					
Control	388 ± 37	170 ± 15	218 ± 38	2.27 ± 0.42	118 ± 15
AZ	249 ± 19*	78 ± 19*	171 ± 22	1.96 ± 0.53	162 ± 60
PLP	431 ± 50	191 ± 44	240 ± 27	1.87 ± 0.57	230 ± 50
Tris	439 ± 32	234 ± 38	205 ± 27	2.05 ± 0.33	139 ± 31
Van	310 ± 22	166 ± 23	144 ± 7	1.86 ± 0.22	91 ± 11
CCA[1]					
Control	14 ± 1	12 ± 1	2.0 ± 0.3	0.06 ± 0.01	34 ± 5
AZ	7 ± 1*	5 ± 1*	2.4 ± 0.9	0.05 ± 0.02	61 ± 20
PLP	13 ± 2	7 ± 2	5.7 ± 1.3*	0.07 ± 0.01	143 ± 64
Tris	12 ± 2	10 ± 1	2.6 ± 0.7	0.05 ± 0.00	48 ± 13
Van	13 ± 3	11 ± 2	2.5 ± 1.0	0.08 ± 0.02	29 ± 6
Halimeda opuntia					
Control	117 ± 12	56 ± 10	61 ± 5	0.71 ± 0.28	174 ± 34
AZ	95 ± 12	21 ± 5*	74 ± 7	0.28 ± 0.05	372 ± 38*
PLP	122 ± 9	47 ± 6	75 ± 6	0.51 ± 0.04	186 ± 22
Tris	117 ± 14	51 ± 11	66 ± 5	0.38 ± 0.15	284 ± 49
Van	92 ± 6	34 ± 5	58 ± 6	0.40 ± 0.08	196 ± 29
Jania adhaerens					
Control	550 ± 36	476 ± 37	74 ± 12	1.27 ± 0.18	60 ± 10
AZ	188 ± 21*	84 ± 11*	104 ± 17	1.73 ± 0.44	91 ± 23
PLP	329 ± 46*	278 ± 52*	51 ± 9	1.21 ± 0.14	50 ± 11
Tris	481 ± 31	432 ± 36	49 ± 9	1.01 ± 0.06	48 ± 9
Van	503 ± 52	435 ± 52	68 ± 6	1.32 ± 0.16	57 ± 8
Laurencia intricata					
Control	504 ± 32	397 ± 30	107 ± 9	0.86 ± 0.06	130 ± 12
AZ	342 ± 21	199 ± 19	144 ± 12	0.88 ± 0.09	185 ± 19
PLP	325 ± 65*	156 ± 61*	169 ± 22*	0.81 ± 0.19	240 ± 61
Tris	280 ± 23*	112 ± 15*	168 ± 24*	1.06 ± 0.19	215 ± 75
Van	292 ± 48*	180 ± 48	112 ± 8	1.00 ± 0.08	127 ± 14
Neogoniolithon strictum					
Control	183 ± 11	115 ± 13	68 ± 5	0.28 ± 0.03	297 ± 50
AZ	191 ± 28	111 ± 18	79 ± 15	0.34 ± 0.06	275 ± 62
PLP	174 ± 16	100 ± 15	74 ± 8	0.28 ± 0.04	326 ± 68
Tris	165 ± 9	100 ± 8	65 ± 5	0.31 ± 0.03	237 ± 27
Van	168 ± 19	112 ± 16	56 ± 5	0.27 ± 0.03	218 ± 12

Sargassum fluitans					
Control	571 ± 65	414 ± 66	157 ± 12	2.69 ± 0.41	72 ± 15
AZ	153 ± 20*	-27 ± 6*	180 ± 14	1.99 ± 0.59	-
PLP	400 ± 57	265 ± 64	135 ± 12	2.07 ± 0.26	83 ± 14
Tris	374 ± 33*	155 ± 20	219 ± 30	3.35 ± 0.67	87 ± 15
Van	481 ± 67	309 ± 57	172 ± 13	3.48 ± 0.47	57 ± 6
Udotea luna					
Control	608 ± 101	278 ± 59	330 ± 48	2.91 ± 0.48	135 ± 7
AZ	365 ± 41*	-27 ± 022*	392 ± 57	3.56 ± 1.05	223 ± 24
PLP	524 ± 55	158 ± 51	366 ± 44	2.99 ± 0.61	155 ± 20
Tris	416 ± 41	12 ± 25*	404 ± 56	2.88 ± 0.57	263 ± 21*
Van	646 ± 62	310 ± 44	336 ± 27	2.48 ± 0.55	203 ± 39

Table S6. Two-way ANOVA table for the combined effects of pH and AZ on gross photosynthesis maxima (P_{gmax}) for *C. cervicornis and J. adhaerens.* Significant effects ($p < 0.05$) are marked with an asterisk.

	df	Sum sq	Mean sq	F-value	p-value
Canistrocarpus cervicornis					
AZ	1	0.3363	0.3363	31.282	6.91e-07*
pH	3	1.4517	0.4839	45.004	6.15e-15*
AZ x pH	3	0.0327	0.0109	1.013	0.394
Jania adhaerens					
AZ	1	0.19462	0.19462	23.631	3.2e-05*
pH	3	0.10139	0.03380	4.104	0.0146*
AZ x pH	3	0.03562	0.01187	1.442	0.2496
